# Combining analytical epidemiology and genomic surveillance to identify risk factors associated with the spread of antimicrobial resistance in *Salmonella enterica* subsp. *enterica* serovar Heidelberg

**DOI:** 10.1099/mgen.0.000891

**Published:** 2022-11-23

**Authors:** Benjamin M. Hetman, David L. Pearl, Dillon O. R. Barker, James Robertson, John H. E. Nash, Richard Reid-Smith, Agnes Agunos, Catherine Carrillo, Edward Topp, Gary Van Domselaar, E. Jane Parmley, Amrita Bharat, Michael Mulvey, Vanessa Allen, Eduardo N. Taboada

**Affiliations:** ^1^​ Department of Population Medicine, Ontario Veterinary College, University of Guelph, Guelph, Ontario, Canada; ^2^​ National Microbiology Laboratory, Public Health Agency of Canada, Winnipeg, Manitoba, Canada; ^3^​ National Microbiology Laboratory, Public Health Agency of Canada, Guelph, Ontario, Canada; ^4^​ Centre for Foodborne, Environmental and Zoonotic Infectious Diseases, Public Health Agency of Canada, Guelph, Ontario, Canada; ^5^​ Ottawa Laboratory (Carling), Canadian Food Inspection Agency, Ottawa, Ontario, Canada; ^6^​ London Research and Development Centre, Agriculture and Agri-Food Canada, London, Ontario, Canada; Department of Biology, University of Western Ontario, London, Ontario, Canada; ^7^​ Public Health Ontario Laboratory, Toronto, Ontario, Canada; ^†^​ Present address: Department of Laboratory Medicine and Pathobiology, University of Toronto, Ontario, Toronto, Canada

**Keywords:** antimicrobial resistance, genomic epidemiology, salmonella, surveillance, whole genome sequencing

## Abstract

Antimicrobial resistance (AMR) has become a critical threat to public health worldwide. The use of antimicrobials in food and livestock agriculture, including the production of poultry, is thought to contribute to the dissemination of antibiotic resistant bacteria (ARB) and the genes and plasmids that confer the resistant phenotype (ARG). However, the relative contribution of each of these processes to the emergence of resistant pathogens in poultry production and their potential role in the transmission of resistant pathogens in human infections, requires a deeper understanding of the dynamics of ARB and ARG in food production and the factors involved in the increased risk of transmission.

## Full-Text

For this study, we analysed the whole genome sequence (WGS) of 430 isolates of *Salmonella enterica* subsp. *enterica* serovar Heidelberg from surveillance of poultry from various points of the poultry production pyramid (broiler farms at chick placement and pre-harvest/pre-shipment stage), abattoirs and from retail/grocery stores, as well as passively collected poultry isolates from diagnostic submissions and human clinical submissions in Ontario, Canada in 2013. Genome assemblies were subjected to *in silico* AMR detection and the population structure was assessed using a novel *S*. Heidelberg-specific 2358-gene core genome Multi-Locus Sequence Typing assay and core-genome single nucleotide variant analysis. We then used multilevel logistic regression to explore associations between genetic determinants of resistance, bacterial population structure, and factors related to poultry production.

Our results are consistent with the possible transmission of *S*. Heidelberg between poultry sources and humans and support previous work suggesting that *S*. Heidelberg in Canada comprises a diverse genomic population, with the dissemination of antimicrobial resistance likely due to the presence of highly mobile plasmids conferring the resistant phenotype. Our risk factor analysis showed that isolates sampled from chicken were at over ten-fold increased odds of carrying the AmpC beta-lactamase *bla*_CMY-2_ compared to turkey associated isolates. Moreover, the odds of carrying the ColRNAI plasmid replicon type were higher in later stages of poultry production.

As genome sequencing datasets from public health surveillance continue to grow, optimizing the application of analytical approaches that leverage both high resolution genetic data and underlying epidemiologically relevant information collected at the point of sampling will become increasingly important. Taken together, the methodology and results of our analysis may serve to inform future studies that seek to elucidate the transmission dynamics of AMR from surveillance and WGS data and inform policy on suitable areas for intervention to help reduce the burden of AMR on human health.

## Data Summary

Please see the data bibliography for supporting data and analyses. All data used in this manuscript were provided to the researchers by the Canadian Integrated Program for Antimicrobial Resistance Surveillance. For additional information about Canadian antimicrobial resistance surveillance protocols, requests can be made via email to phac.cipars-picra.aspc@phac-aspc.gc.ca. No information that could be used to identify humans, farms, abattoirs, or other facilities from which bacterial isolates were sampled was provided to the authors of the study. All supporting genomic sequences used in this analysis have been deposited in the NCBI Sequence Read Archive under BioProject accession number PRJNA845137.

**Impact Statement**

As WGS data from pathogen surveillance becomes increasingly available, it becomes critical to leverage these data for maximum public health impact. In this study, we show that *S*. Heidelberg represents a complex population, with widespread third-generation cephalosporin resistance likely due to its association with a highly mobile IncI1 plasmid. Additionally, our models predict that association of this resistance is over ten-fold higher when assessing isolates from chicken compared to turkey. These results provide additional weight to the body of evidence that broiler chicken production plays an important role in the dissemination of resistant strains of *S*. Heidelberg and the plasmid families that confer AMR. An important aspect of our study was the application of principles adapted from analytical epidemiology including considerations for sample size, confounding factors, and repeatability. Notably, although this imposed limitations on the number/types of analyses possible, it improved the reliability and robustness of our conclusions, a critical consideration as we transition from a paradigm in which genomic epidemiology is primarily used as a tool to investigate specific public health events and towards the translation of genomic surveillance data into public health policy.

## Introduction

Antimicrobial resistance (AMR) is a critical public health issue, with forecasts predicting up to ten million deaths per year related to AMR by 2050 [1]. The use of antimicrobials in food animal production, including poultry, is thought to contribute to the dissemination of antibiotic resistant bacteria (ARB) and the genes and plasmids that confer the resistant phenotypes (ARG). However, determining the true extent of ARB/ARG transmission to humans from food animal production and the contribution of these processes to rising rates of human infections with resistant pathogens requires a deeper understanding of the dynamics of ARB and ARG in food animal production systems, and the factors that impact their risk of transmission [2, 3].

Two primary mechanisms are responsible for the dissemination of acquired resistance to antimicrobials in a bacterial population: the clonal dissemination of a strain possessing acquired ARG, and lateral gene transfer events which can be largely mediated by mobile genetic elements (MGE) such as plasmids [4–6]. Uncovering the mechanisms responsible for transmission of AMR in a bacterial population requires knowledge of both the population structure, and the presence of transmissible elements related to AMR. Recently, whole-genome sequencing (WGS) has become increasingly deployed in routine pathogen surveillance. With sequencing costs having decreased substantially over the past two decades [7], WGS has become a cost-effective method to replace gold-standard laboratory techniques for the characterization of bacterial isolates, including high-resolution subtyping data for tracking pathogen strain types in circulation and providing detailed information on important features associated with increased risk to public health [8–10]. Along with the increasing availability of WGS data, several bioinformatics tools and pipelines have been developed for the analysis of population structure (e.g., *chewBACCA*, Grapetree, SNVPhyl) and AMR (e.g., CARD, Resfinder, ARIBA), many of which do not have high-performance computing requirements [11–15]. Together, the concomitant availability of WGS data and the computational tools needed to analyse them have the potential to revolutionize AMR surveillance.

In light of these advances, we sought to apply WGS to isolates of *Salmonella enterica* subsp. *enterica* serovar Heidelberg using methods from an ‘analytical epidemiology’ framework (i.e., an epidemiological approach focused on measuring associations between a health condition and exposures) [16] to explore factors important to the transmission of AMR in poultry production. Isolates of *S*. Heidelberg are often resistant to antimicrobials, notably third generation cephalosporins, and this serovar is consistently reported among the most frequently isolated *Salmonella* serovars from human infections across Canada [17], as well as from samples collected from surveillance in various stages of the poultry production continuum [18]. Moreover, human infections with *S*. Heidelberg are frequently invasive, and *S*. Heidelberg often demonstrates resistance to clinically relevant third-generation cephalosporins, such as ceftriaxone, which is associated with the extra-label use of cephalosporins in poultry production to prevent certain bacterial infections such as avian pathogenic *E. coli* infections [19, 20]. Few genetic differences appear to exist between *S*. Heidelberg that pose a risk to human health and those that are frequently isolated serovars from the chicken intestines and their environment (barns and slaughterplants), restricting the usefulness of traditional molecular methods, and limiting our understanding of the epidemiology of this important pathogen [21, 22].

To explore factors important to the transmission of AMR in *S*. Heidelberg from poultry production in Ontario, Canada, we applied both genomic analysis and epidemiologically informed statistical modelling to a collection of 430 *S*. Heidelberg genome sequences of isolates collected from various stages of the poultry production continuum and human clinical samples in Ontario, Canada. This paper describes the presence of ARG and MGE related to the transmissibility of AMR, as well as the population structure determined by a novel core-genome multi-locus sequence typing schema (cgMLST) specific for *S*. Heidelberg and validated using core-genome single nucleotide variant (SNV) analysis. Results from the genomic analysis were used in multilevel logistic regression models to examine associations between stages of poultry production, poultry type, population structure, and the carriage of ARG and MGE while controlling for confounding effects related to surveillance-based sampling within the poultry production system.

## Methods

### Sample collection

Isolates of *S*. Heidelberg used in this study were collected through the Canadian Integrated Program for Antimicrobial Resistance Surveillance (CIPARS) [23] and the Canadian Food Inspection Agency (CFIA) poultry microbiological baseline study [24] in 2013. Briefly, CIPARS actively samples broiler chickens from farms and abattoirs as well as chicken meats at retail/grocery stores and collects *S*. Heidelberg isolates from human clinical samples through a passive submission process. Of note, chicken samples collected from ‘hatchery’ stage are in fact meconium collected from chick pads immediately after arrival of the chicks at the barn and are considered an appropriate representation for earlier production such as broiler breeder and hatchery stage. Hatchery samples from turkey were collected as part of non-routine sampling of environmental swabs or fluff samples.

**Fig. 1.**
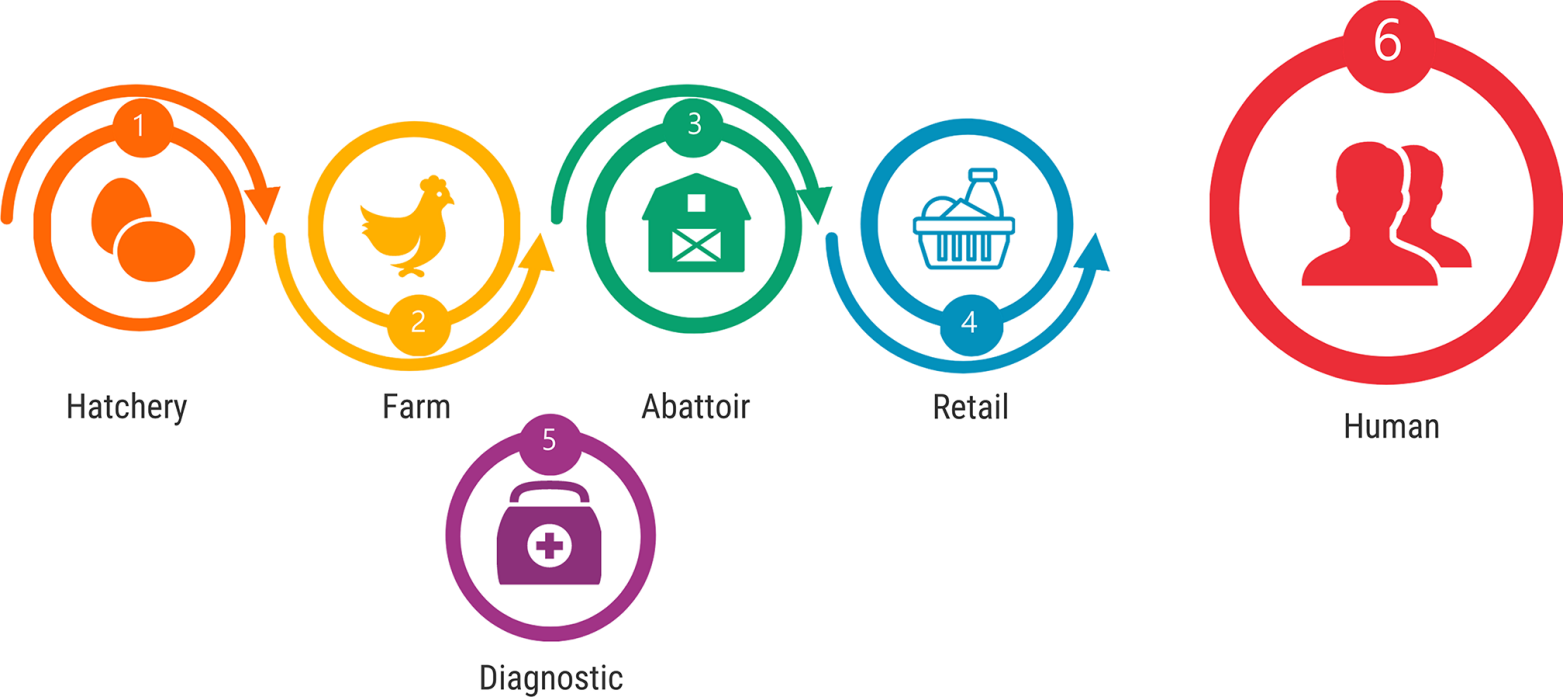
Samples collected from poultry production stages and routine submissions to AMR surveillance. Stages are numbered in logical order and as used in statistical models. (1) Hatchery, (2) Broiler farm, (3) Abattoir, and (4) Retail samples were collected as part of active sampling initiatives by the Public Health Agency of Canada and the Canadian Food Inspection Agency. Isolates were collected from (5) Diagnostic sources passively, through veterinarian submissions to the CIPARS surveillance system. (6) Human clinical isolates were collected passively through submissions to the Public Health Ontario laboratory through routine notifiable disease surveillance.

Sampling for the CFIA poultry baseline study was designed to closely align with the CIPARS data collection process and provide a valid supplementary source for augmenting CIPARS surveillance data. Information collected alongside samples included data on source of isolation (e.g., sampled bird species, location, sample matrix) and collection date, as well as phenotypic antimicrobial resistance test results from standardized antibiograms performed by CIPARS for its surveillance isolates and the CFIA baseline isolates [23]. For this study we chose to restrict our research focus to isolates collected from CIPARS and the CFIA poultry baseline study in Ontario, Canada from 2013 in order to maximize the numbers of isolates collected from a consistent location and time and minimize inconsistencies in the sample metadata related to province and year of sampling. This timing also coincided with the first year for active on-farm sampling for CIPARS, and represents the final year before the implementation of the poultry industries antimicrobial use reduction strategy [25]. *Salmonella* isolates from human clinical submissions in [26] were also included for comparison in this study; these isolates were collected by the Public Health Ontario Laboratory (PHOL) as part of routine reportable enteric disease surveillance. Isolates were cultured from blood, stool, and urine specimens and subjected to antimicrobial susceptibility testing and whole-genome sequencing by PHOL, or by the National Microbiology Laboratory in Winnipeg, MB (Fig. 1).

### Whole-genome sequencing and genome assembly

Preparations of high-quality DNA extractions were performed at the National Microbiology Laboratory (NML) in Guelph, ON, and Winnipeg, MB, and were either sequenced on-site or distributed to partnering laboratories at the CFIA (Ottawa, ON; Calgary, AB; Sherbrooke, QC; Burnaby, BC) for sequencing using Illumina MiSeq and NextSeq platforms. Raw genome sequences were uploaded to the Integrated Rapid Infectious Diseases Analysis platform (https://irida.corefacility.ca/) at the NML in Winnipeg, MB, for quality assessment and data storage. Raw reads were assembled using the Spades assembler [27] as part of the *Shovill* pipeline (https://github.com/tseemann/shovill) with the following optional settings applied: ‘--minlen 200 --mincov 2 --depth 60 --gsize 4.8M; --assembler spades; --trim’. Genome assemblies were deposited online at the NCBI Sequence Read Archive under BioProject accession number PRJNA845137.

### Analysis of whole-genome assemblies

#### AMR prediction

Genome assemblies were queried for acquired resistance genes using the AMR prediction tool *StarAMR* (v.0.7.2) (https://github.com/phac-nml/staramr) and the *Resfinder* database [28] containing definitions current as of 17 March 2022. Settings included nucleotide coverage of 90 % and percent identity of 98 %. The nucleotide-identity cutoff was increased to 100 % for the prediction of acquired beta-lactamases in order to capture single-nucleotide differences resulting in a change to the allelic determination of the beta-lactamase genes.

#### Plasmid replicon prediction

Plasmid replicon types were assessed using the software tool *MOB-Suite* [29]. Briefly, clusters of similarity groups are established using MASH distances [30] and plasmid sequences are reconstructed from draft genome assemblies using the clustered plasmid reference databases and the *Circlator* assembly tool to identify contigs with overlapping ends [31]. Putative plasmid sequences are then queried against a high-quality curated database to provide predictions of the replicon family, relaxase type, and predicted transferability classification (e.g., conjugative, mobile, non-mobile).

#### Creation of a core-genome multi-locus sequence typing (cgMLST) scheme for *S.* Heidelberg

A novel core-genome Multi-locus Sequence Typing (cgMLST) schema was created for *S*. Heidelberg in this study. The 430 *S*. Heidelberg genome assemblies were supplemented with additional genomes sequenced through Canadian baseline and surveillance programs, and combined with those publicly available at the National Centre for Biotechnology Information (https://www.ncbi.nlm.nih.gov/sra). In total, 4509 genome assemblies of *S*. Heidelberg were available for the creation of the cgMLST scheme. Briefly, genome assemblies were subjected to analysis using the *chewBACCA* pipeline [11], which identifies high-quality gene sequences common to all genomes in the dataset, filters length-variable and paralogous genes, and evaluates the subsequent allele-calling. For inclusion into the cgMLST scheme, we used a strict criterion requiring that loci be present in a minimum of 99.9 % (*n*=4504/4509) of the total genomes queried. A complete description of the cgMLST schema creation, as well as the allelic database resulting from analysis of the 4509 publicly available genomes, is publicly available at https://github.com/dorbarker/salmonella-heidelberg-cgmlst.

#### Core-genome single nucleotide variant (SNV) analysis

The Harvest Tools software package, including the core-genome variant pipeline *parSNP* (version 1.2) [32], was used to determine the core genome SNVs present in our sample. The closed *S*. Heidelberg genome AMR588-04-00435 (accession: NZ_CP016565.1) was used as the reference sequence for the analysis, as the isolate was sampled from similar conditions to those in our study, and therefore provided a closely-related genome for comparison to ensure high discriminatory power (i.e., the ability of a typing system to discriminate between individual bacterial isolates [33]) in the SNV analysis [34, 35]. The optional flags ‘*-c*’ and ‘*-x*’ were used to prevent the exclusion of genomes in the final alignment with maximal unique match indices (MUMi) greater than 0.01, and to filter regions of recombination using the PhiPack tool, respectively [36].

#### Visualization of population structure and optimal cluster determination

Allelic determinations from the cgMLST analysis were clustered using the ‘MSTreeV2’ algorithm and visualized as a minimum spanning tree as part of the stand-alone software package *GrapeTree* (v.1.5) [12]. Cluster definitions for each genome were extracted from the tree at a variety of distance thresholds using a custom python script (available at https://github.com/dorbarker/grapetree_cluster) and were then used to compare the discriminatory power of each clustering threshold. Simpson’s Index of Diversity (SID) is a common diversity index that measures the probability that two individuals selected at random from the population will belong to distinct types, or as used in the present study, clusters [37, 38]. The SID was computed for each clustering threshold by changing the number of different cgMLST loci (*k*) permitted in the same cluster, starting with no allowable differences (i.e., *k*=0) considered for inclusion into the same cluster up to a maximum of *k*=35, where all genomes in the sample formed a single cluster. To evaluate whether cgMLST clusters were generating genetically distinct groups, we employed a Monte Carlo sampling approach seen previously [39], where the mean genomic distance within and between each of the defined cgMLST clusters (measured by the average percent difference of core SNVs) was compared to equal-sized, randomly-selected clusters of genomes from the sample population. The size of the randomly selected groups was kept consistent with the number of genomes for each comparison cluster, and 9999 iterations of random sampling were used to create a distribution of mean similarities for hypothesis testing. The mean genomic distances for each cluster from the cgMLST analysis was calculated using the ‘*clv*’ package in R, then ranked relative to the distribution of mean distances from the randomly sampled isolates to estimate a *P*-value.

### Construction of statistical models

The origin of sample collection was manually assessed and one of five aggregate terms describing poultry production stages was applied for each bacterial isolate from turkey and chicken sources. These terms included ‘hatchery’ for samples collected upon entry of birds to the broiler farm; ‘broiler farm’ for samples collected from birds approximately 1 week prior to shipment for slaughter; ‘abattoir’ for samples collected from the caecal content of bird carcasses; ‘retail’ for meat samples collected from consumer grocery outlets; and ‘diagnostic/clinical’ for samples collected passively from sick animals and submitted to specialized veterinary and microbiological laboratories for culturing and analysis. Isolates collected from human clinical specimens were designated ‘human’ samples, regardless of sample matrix (e.g., ‘blood’, ‘stool’, ‘rectal swab’). A field representing clustering at the establishment level was constructed to control for clustering of poultry isolates actively sampled through surveillance from the same location (e.g., poultry barn, abattoir, retail store) on the same date in subsequent statistical analyses. Isolates sampled from the identical production stage and date were assumed to have been sampled from the same establishment. This designation was verified where possible using available information including project codes and anonymized institution identifiers. Isolates sampled on unique combinations of date and location and lacking any additional details from collection by surveillance were considered as individual observations in the statistical models. All isolates of human origin and from diagnostic veterinary samples were not considered to be clustered for the statistical analysis and were treated as individual observations for multilevel models.

Univariable multilevel logistic regression models were fitted in Stata v14.2 [40] using the ‘*meqrlogit’* command to explore associations between the presence of genetic determinants related to AMR (i.e., dependent variable) in isolates sampled from poultry (e.g., presence of acquired genes and plasmids determined from bioinformatic analyses) and the following categorical independent variables: poultry production stage, bird species, and the six largest cgMLST clusters identified from the assessment of *S*. Heidelberg population structure. For the cgMLST cluster variable, observations from both poultry and human isolates were examined. Both AMR determinants and plasmid results from bioinformatic analyses were screened-in for analysis if they were present in 10–90 % of the isolates examined; determinants outside of this frequency range were excluded from statistical analyses to ensure an adequate effective sample size for logistic regression modelling. A Phi coefficient was used to determine if dichotomous independent variables were significantly correlated; if correlation was assessed to be greater than |0.75| the variables were not included in the same model to avoid issues with collinearity. Only genomes included in the largest six cgMLST clusters (each with *n* >9 isolates) identified from the population structure analysis were included in the regression models to improve statistical power and convergence of the regression models. After the construction of each model, the intraclass correlation coefficient was estimated in Stata using the function ‘*estaticc*’ (see script ON13.SH430.PUBLISH.do in [26]) to assess the correlation among samples within the same establishment [16].

**Fig. 2.**
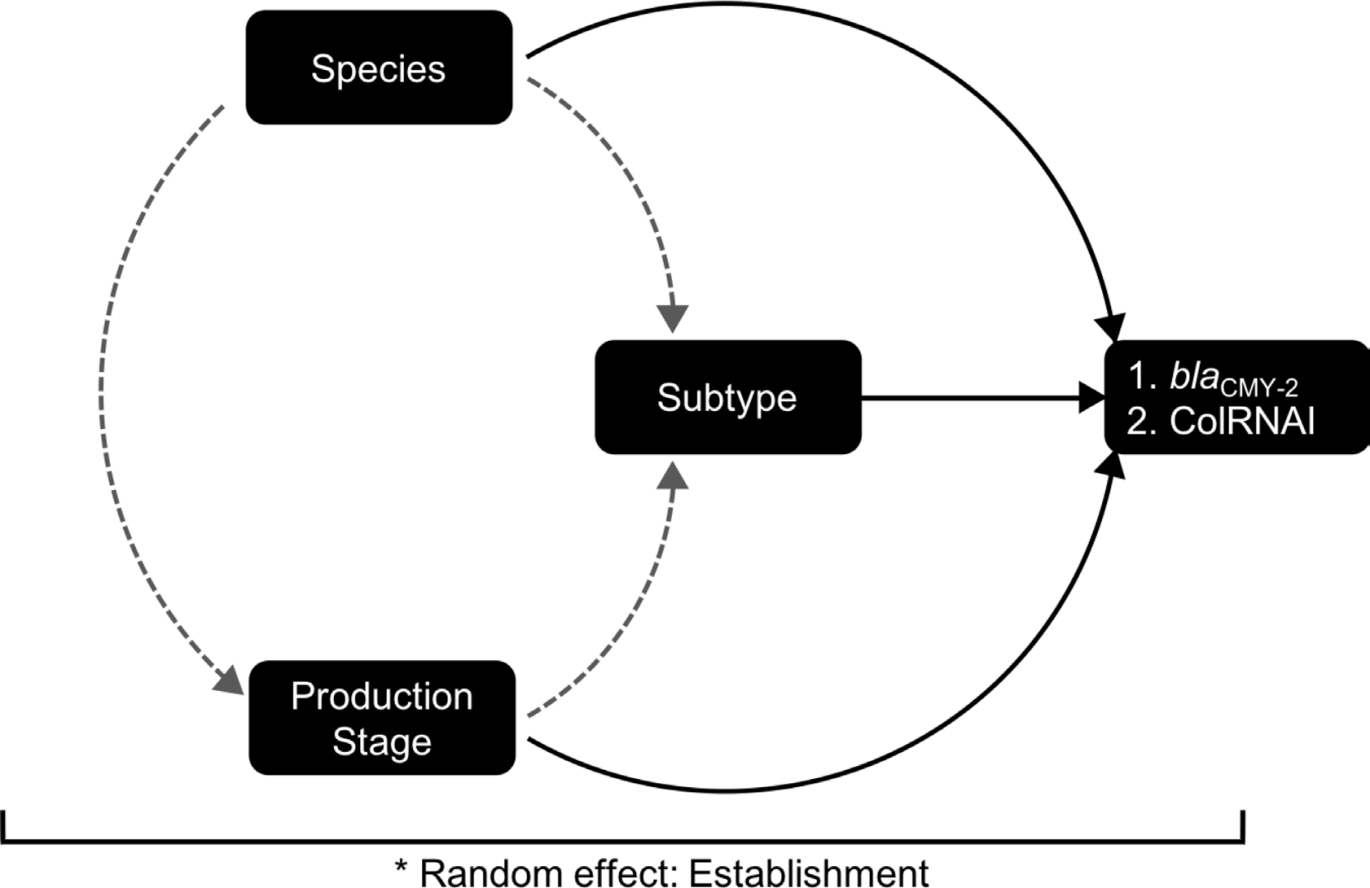
Causal diagram to inform the structure of epidemiologic modelling. Solid lines show direction of proposed univariable relationships between independent and dependent variables. Dashed grey lines show potential confounding relationships.

If an independent variable was statistically significant in a univariable model based on a liberal *P*-value (i.e., alpha=0.2), it was considered for multivariable modelling. A variable could be included in a final model if it was statistically significant (alpha=0.05) or was an explanatory antecedent (i.e., a confounding variable). A variable was considered an explanatory antecedent if its removal resulted in a 30 % or greater change to a statistically significant model coefficient and it was not an intervening variable (i.e., causally located between the variable of interest and the outcome) [16]; a causal diagram was used to differentiate explanatory antecedents from intervening variables (Fig. 2). Univariable models were presented if no additional variables were statistically significant or were explanatory antecedents. Model fit was assessed for each of the final models by visually evaluating the assumptions of normality and homoskedasticity of the best linear unbiased predictors (BLUPs, these may also be referred to as the predictions of the random effects in a mixed model) and evaluating Pearson residuals for the presence of outliers. If a plot of the BLUPs versus the linear predictions of the fitted model showed a band of points with no evidence of fanning (i.e., increasing dispersion of BLUPs as the predicted outcome increases) or coning (i.e., decreasing dispersion of BLUPs as the predicted outcome increases) and the normal quantile plot of the BLUPs fell on a 45 degree line, the assumptions of homoskedasticity and normality, respectively, were considered to have been met [16]. The intra-class correlation coefficient (ICC), an estimate of the correlation in the outcome among individuals within the same establishment, was estimated using the latent variable technique [16, 41].

## Results

### Description of dataset

A total of 430 isolates of *S*. Heidelberg were selected for inclusion in this study. The majority (*n*=279/430; 64.9 %) of isolates were collected from broiler chickens, followed by turkeys (*n*=107/430; 24.9 %), and finally human sources (*n*=44/430; 10.2 %). Broiler chicken-related isolates were predominantly sampled from farms (*n*=98/279; 35.1 %), followed by retail (*n*=92/279; 33 %), hatchery (*n*=50/279; 17.9 %), diagnostic/clinical (*n*=22/279; 7.9 %), and abattoir (*n*=17/279; 6.1 %) sources. By contrast, isolates from turkey production were predominantly sampled from hatchery (*n*=85/107; 79.4 %), followed by retail (*n*=13/107; 12.1 %), turkey farm (*n*=4/107; 3.7 %), and diagnostic/clinical (*n*=5/107; 4.7 %) sources. Isolates collected from human sources were predominantly sampled from stool (*n*=37/44; 84.1 %), followed by blood (*n*=3/44; 6.8 %), urine (*n*=3/44; 6.8 %), and from the rectum by rectal swab (*n*=1/44; 2.3 %) (Table 1).

**Table 1.** Counts and proportions by production level and bird species for *Salmonella* Heidelberg isolates collected from poultry and human sources in Ontario, Canada, 2013

Production Stage	Chicken (%)	Turkey (%)	Total (%)
Hatchery*	50 (18)	85 (79)	**135** (31)
Farm	98 (35)	4 (4)	**102** (24)
Diagnostic/Clinical	22 (8)	5 (5)	**26** (6)
Abattoir	17 (6)	–	**17** (4)
Retail	92 (33)	13 (12)	**105** (25)
Human	–	–	**44**(10)
**Total**	**279** (65)	**107** (25)	**430**

*Chick pads and environmental swabs sampled at arrival on-farm; used as a surrogate for hatchery samples.

### *In silico* AMR and plasmid determination

Genome sequences were queried for the presence of known acquired resistance genes. In total, 31 genetic determinants of AMR were identified in our dataset, representing acquired resistance to seven different classes of antimicrobials. The most common determinant was *fos*A7, conferring resistance to fosfomycin, with 423/430 genomes (98.4 %, 95 % CI: 96.7–99.2 %) positive for carriage. Among the 430 genomes examined, 49 isolates carried genes for resistance to aminoglycosides (11.4 %, 95 % CI: 8.6–14.7 %), 174 carried genes for resistance to beta-lactams (40.4 %, 95 % CI: 35.9–45.2 %), 33 carried genes for folate pathway inhibition (7.7 %, 95 % CI: 5.4–10.5 %), 27 carried genes conferring resistance to tetracyclines (6.3 %, 95 % CI: 4.2–9.0 %), and four carried genes conferring phenicol resistance (0.9 %, 95 % CI: 0.3–2.3 %). Three isolates were found to carry the aac(6’)-Ib-cr determinant (0.7 %, 95 % CI: 0.2–1.9 %), which can induce resistance to both aminoglycosides and fluoroquinolones simultaneously. Colistin resistance was detected in three isolates (0.7 %, 95 % CI: 0.2–1.9 %), and the sitABCD operon conferring resistance to oxidative stress was detected in five isolates (1.1%, 95 % CI: 0.4–2.7 %). No genes were found that confer acquired resistance to macrolides or carbapenems, and resistances conferred by chromosomal mutations were not examined (Table 2).

**Table 2.** Frequencies of antimicrobial resistance genes identified using whole-genome sequencing data of *Salmonella* Heidelberg isolates collected from poultry and human sources, Ontario, Canada 2013

Antimicrobial group	Resistance gene	Accession no.*	Count	(% of 430)
Aminoglycoside	*aac*(3)-IId	EU022314	7	(1.6 %)
	*aac*(3)-VIa	NC_009838	20	(4.7 %)
	*aac*(6')-Ib-cr†	DQ303918	3	(0.7 %)
	*aac*(6')-Ib3	X60321	3	(0.7 %)
	*aad*A1	JQ414041	9	(2.1 %)
	*aad*A2	JQ364967	3	(0.7 %)
	*ant*(3'')-Ia	X02340	28	(6.5 %)
	*aph*(3')-Ia	EU722351	2	(0.5 %)
	*aph*(3'')-Ib	AF321551	10	(2.3 %)
	*aph*(6)-Id	M28829	12	(2.8 %)
	*grd*A	QJX10702	2	(0.5 %)
Beta-lactam	*bla*CMY-2	X91840	156	(36.3 %)
	*bla*TEM-1A	HM749966	5	(1.2 %)
	*bla*TEM-1B	AY458016	12	(2.8 %)
	*bla*TEM-214	KP050491	1	(0.2 %)
Colistin	*mcr*-9	NZ_NAAN01000063.1	3	(0.7 %)
Folate pathway inhibitors	*dfr*A1	AJ238350	1	(0.2 %)
	*dfr*A12	AM040708	3	(0.7 %)
	*dfr*A14	AF393510	1	(0.2 %)
	*dfr*A15	AF221900	1	(0.2 %)
	*dfr*A8	U10186	1	(0.2 %)
	*sul*1	U12338	25	(5.8 %)
	*sul*2	AY034138	5	(1.2 %)
	*sul*3	AJ459418	4	(0.9 %)
Oxidative stress	sitABCD	AY598030	5	(1.2 %)
Phenicol	*flo*R	AF118107	1	(0.2 %)
	*cml*A1	M64556	3	(0.7 %)
Fosfomycin	*fos*A7	LAPJ01000014	423	(98.4 %)
Tetracycline	*tet*(A)	AF534183	20	(4.7 %)
	*tet*(B)	AF326777	6	(1.4 %)
	*tet*(M)	U58985	1	(0.2 %)

*Accession values provided as part of *Resfinder* database, 17 Mar 2022.

†This gene may also confer fluoroquinolone resistance.

All genomes were positive for the carriage of at least one plasmid replicon type. The most commonly identified plasmid type was from Incompatibility group X1 (IncX1), found in 409/430 (94.4 %; 95 % CI: 92.7–96.9 %) of genomes queried. The plasmid types CoIRNAI (*n*=334/430, 95 % CI: 73.5–81.5 %) and IncI1 (*n*=198/430, 95 % CI: 41.3–50.8 %) were also found at high frequencies. Eleven other plasmid types were also present according to *in silico* predictions and represented varying degrees of mobility according to our analysis using MOB-suite software (Table 3). A complete summary table of isolate AMR and plasmid results is available in Table S1.

**Table 3.** Frequencies and predicted mobility of plasmid replicon types identified using whole-genome sequencing data of *Salmonella* Heidelberg isolates collected from poultry and human sources in Ontario, Canada 2013

	Plasmid mobility							
Rep-type	Conjugative (%)		Mobilizable (%)		Non-mobilizable (%)		Total	(% of 430)
IncX1	389	(95.1)	7	(1.7)	13	(3.2)	409	(95.1)
ColRNAI	0	(0)	319	(95.5)	53	(15.9)	334	(77.7)
IncI1	188	(94.9)	9	(4.5)	1	(0.5)	198	(46.0)
IncX4	13	(100)	0	(0)	0	(0)	13	(3.0)
IncFII	7	(100)	0	(0)	1	(14.3)	7	(1.6)
IncFIIA	6	(85.7)	0	(0)	2	(28.6)	7	(1.6)
IncH	2	(33.3)	0	(0)	4	(66.7)	6	(1.4)
IncFIB	5	(100)	0	(0)	0	(0)	5	(1.2)
IncN	2	(66.7)	0	(0)	1	(33.3)	3	(0.7)
IncP	3	(100)	0	(0)	0	(0)	3	(0.7)
IncA/C2	2	(100)	0	(0)	0	(0)	2	(0.5)
IncI2	0	(0)	0	(0)	2	(100)	2	(0.5)
IncY	0	(0)	0	(0)	1	(100)	1	(0.2)
IncQ1	1	(100)	0	(0)	0	(0)	1	(0.2)

### *Salmonella* Heidelberg population structure

**Fig. 3.**
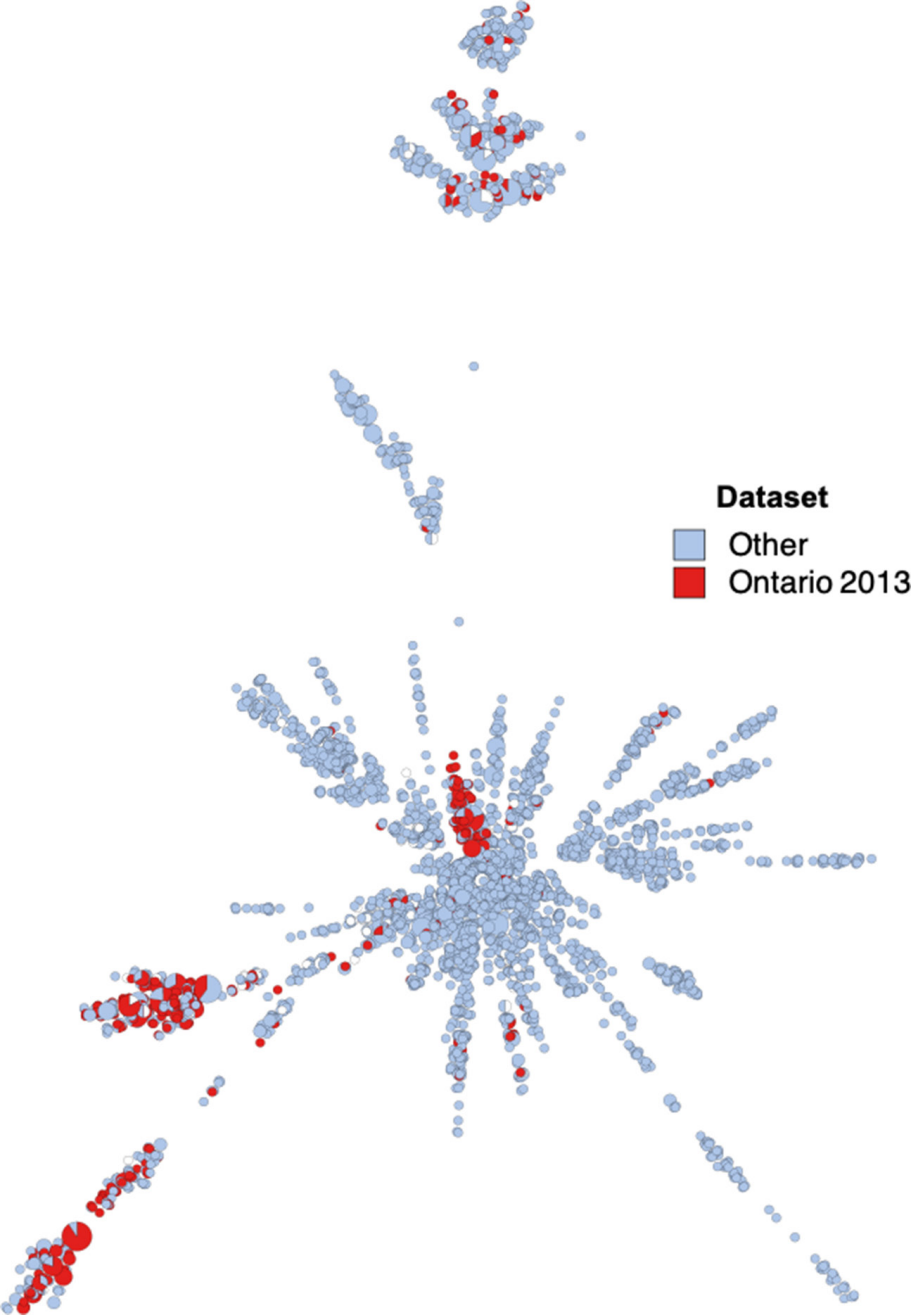
Minimum spanning tree created from novel core genome multi-locus sequence type profiles of 4509 *S*. Heidelberg collected through Canadian surveillance and supplemented with publicly available genomes. Fill colour indicates genomes from global dataset (grey, *n*=4079) and those used for analysis in the current study (red, *n*=430).

A total of 2358 genetic loci were found present in 99.9 % of the combined sample of 4509 public and newly-sequenced *S*. Heidelberg genomes and were included in a cgMLST scheme specific to *S*. Heidelberg; the allelic determinants from this collection were then used to create a minimum spanning tree to derive the population structure of the global dataset and to compare the genetic diversity present in our analysis sample with the overall *S*. Heidelberg population (Fig. 3). A mean of 1.34 (95 % CI: 0.13–2.56) alleles per loci were present in our subset of *S*. Heidelberg (*n*=430), with a minimum of one allele per loci and maximum of five alleles, compared to the global *S*. Heidelberg collection (*n*=4509) which comprised an average of 8.44 (95 % CI: 4.69–12.19) alleles per locus and a range of 1–35 alleles. The genomes from our sample dataset did not appear to be restricted to a single branch on the global tree as they were instead distributed along several regions of the overall *S*. Heidelberg topology, representative of a diverse genetic collection of *S*. Heidelberg.

**Fig. 4.**
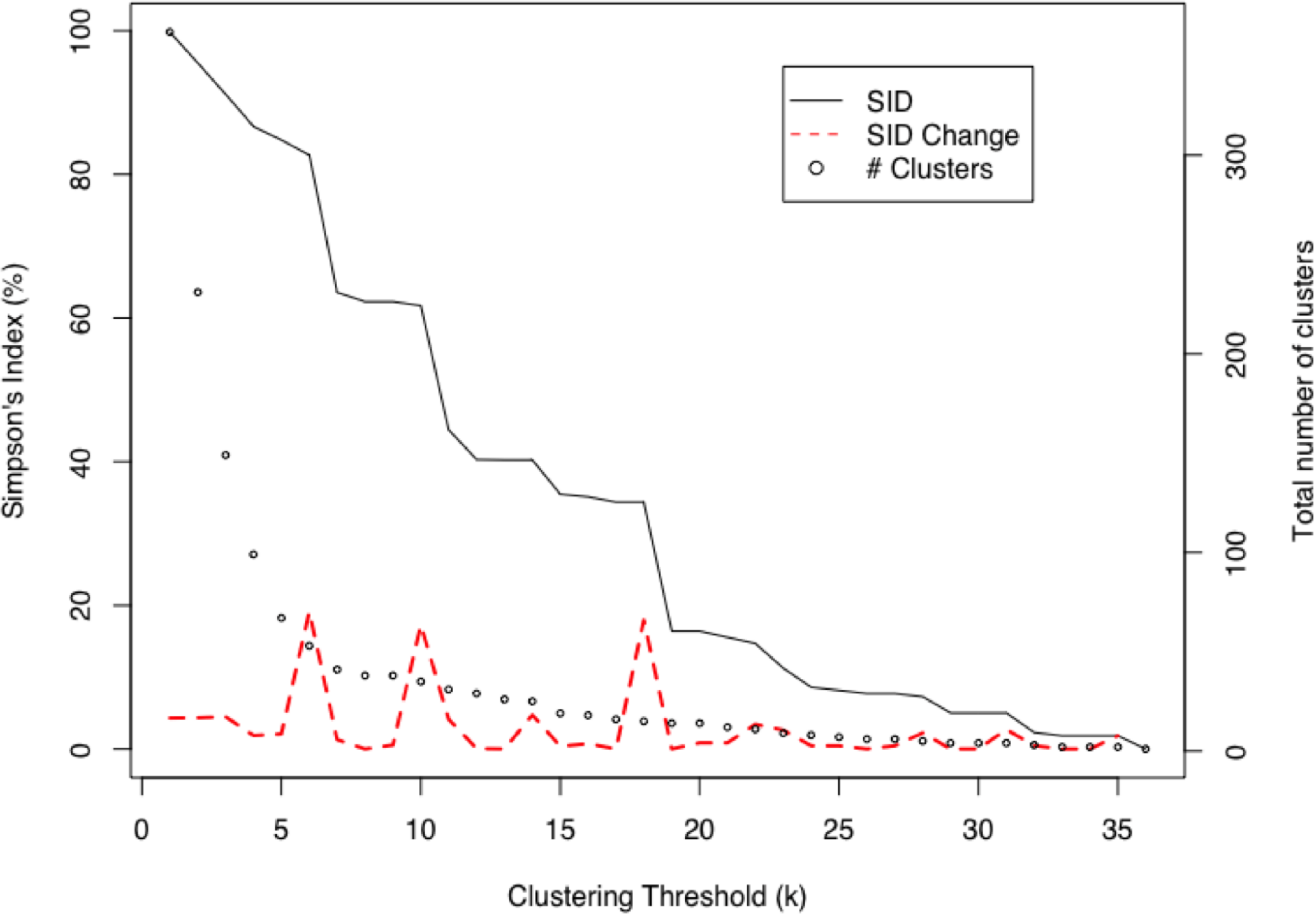
Simpson’s index of diversity (SID) decreases as the cluster threshold (***k***) is relaxed, allowing for larger, more permissive clusters. Dashed red line indicates change in SID as the result of each unit change of *k*. Total number of clusters (including clusters containing single isolate) indicated by dotted black line.

We created a second minimum spanning tree specific to our sample of 430 genomes from Ontario in 2013 in order to test the effects of changing the thresholds used for cluster assignment on the population structure of our sample, and to identify a single threshold to be used in assigning cluster designations for downstream analysis. We calculated Simpson’s index of diversity (SID) for different clustering thresholds to measure changes to the discriminatory power as clustering stringency was relaxed. Starting with a threshold value of *k*=0 (meaning that only genomes with identical allelic profiles are permitted in the same cluster), increasing the allelic threshold values to *k*=6, *k*=10, and *k*=18 (i.e., six, ten, or 18 allelic differences permitted within a single cluster, respectively) prompted the largest changes to SID and sharply reduced the number of total clusters present (Fig. 4). We concluded with a threshold of *k*=5 for our analysis, which produced a total of 53 clusters (SID: 0.827, 95 % CI: 0.804–0.850) with a mean of 13.9 (95 % CI: 10.1–17.8) genomes per cluster on the basis of creating the largest clusters possible without prompting one of the major shifts observed at the previously mentioned thresholds. Of the 53 clusters present at the *k*=5 clustering threshold, 23 comprised multiple genomes, with over 80 % of the dataset (*n*=347/430) distributed within the largest six clusters (Table 4). A supplemental Table S1 (available in the online version of this article) provides a summary of the cgMLST cluster assigned to each isolate, along with *in silico* derived antimicrobial resistance genes, plasmid replicon type and plasmid mobility.

**Table 4.** Optimized (*k*=5) core-genome multi-locus sequence type cluster statistics for *Salmonella* Heidelberg isolates collected from poultry and human sources in Ontario, Canada, 2013

			Mean SNV distance (%)			
cgMLST cluster	n	(%)	Within-cluster	*p* *	Between-cluster	*p* *
4	140	32.5	0.002	< 0.0001	0.008	< 0.0001
18	85	19.8	0.003	< 0.0001	0.008	< 0.0001
1	61	14.2	0.003	< 0.0001	0.008	< 0.0001
27	35	8.1	0.002	< 0.0001	0.009	< 0.0001
20	17	4.0	0.001	< 0.0001	0.007	< 0.0001
22	9	2.1	0.001	< 0.0001	0.009	< 0.0001

* *P*-values based on ranked cluster similarity compared to random distribution of SNV distances generated from Monte Carlo sampling.

**Fig. 5.**
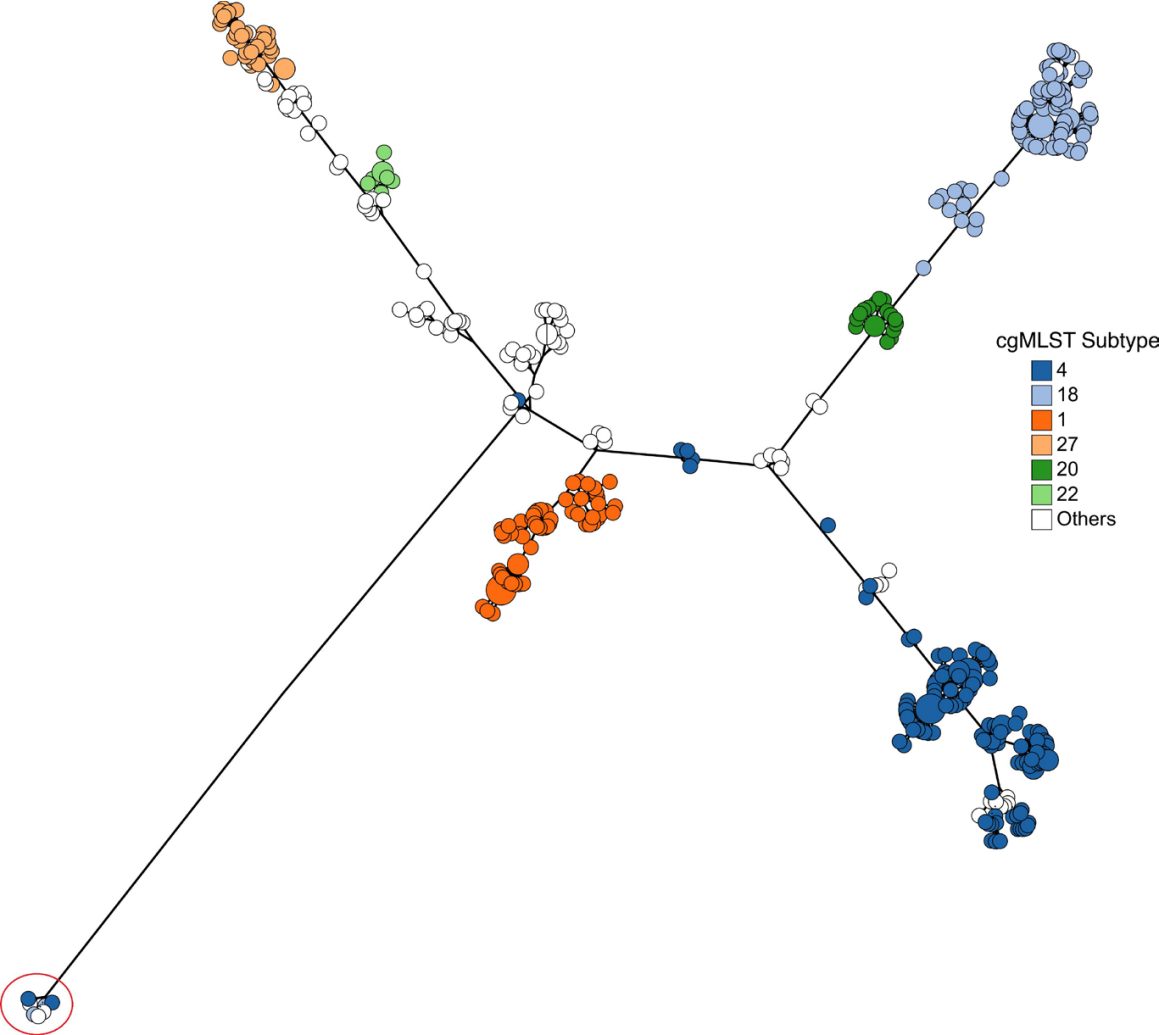
The largest six clusters from cgMLST analysis superimposed on a minimum spanning tree created from core genome SNV results to assess visual concordance between the gene-by-gene and single nucleotide methods. Seven genomes outlined in red represented a distant subgroup in our analysis and were excluded from the calculation of within- and between-cluster distances.

**Fig. 6.**
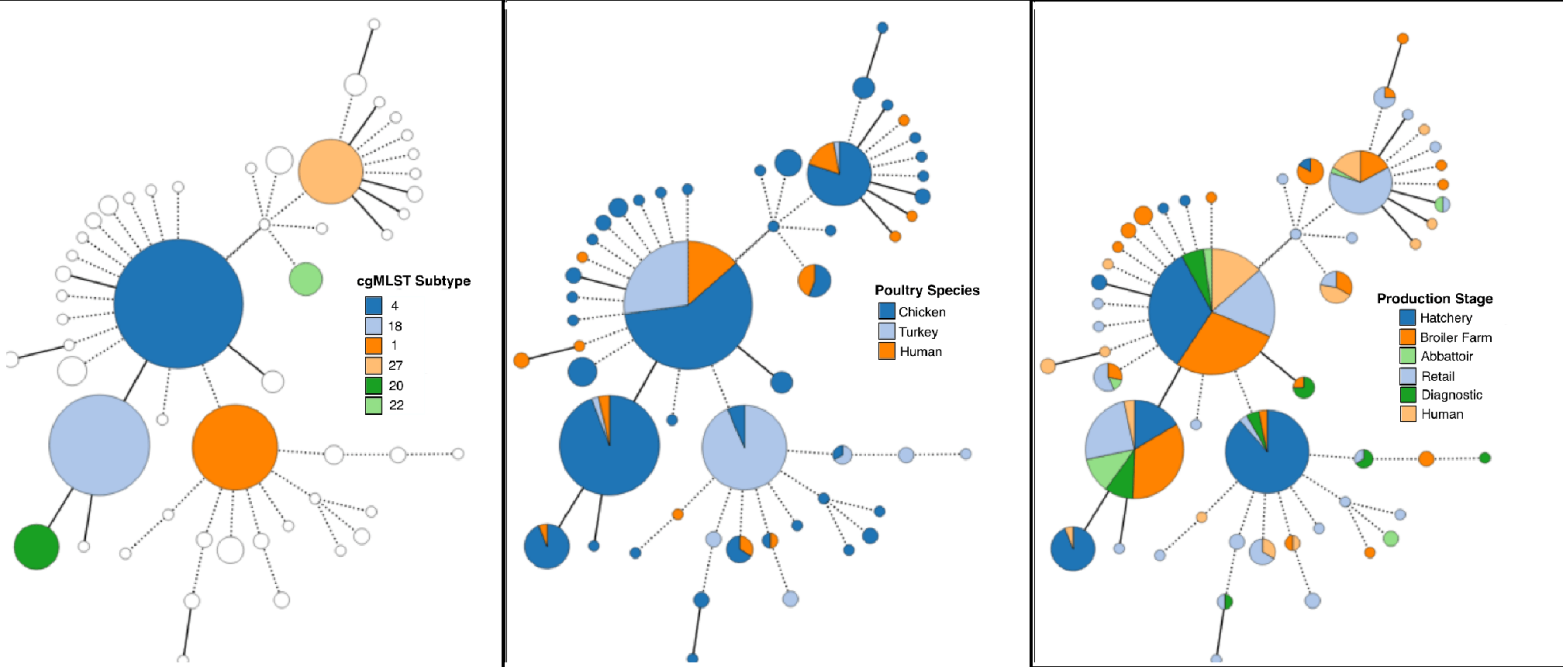
Population structure of 430 *S*. Heidelberg from human and poultry sources in [26]. Minimum spanning trees created using *k*=5 cgMLST clustering threshold. (**a**) Largest six clusters from *k*=5 cluster threshold. (**b**) Distribution of poultry and human *S*. Heidelberg isolates. (**c**) Distribution related to surveillance of poultry production stage or human sampling.

Single-nucleotide variant analysis of the 430 genomes identified seven genomes that appeared to be very distantly related (e.g., > 86 % core SNV-alignment mismatch) to the remainder of the population in our study. We elected to exclude these more distantly related genomes from our subsequent SNV-calculations to facilitate the analysis of core SNV trends among the remaining genomes. Upon exclusion, the number of aligned SNV positions extracted from the core genome was reduced from 50 851 to 7241, with a coverage of 88 % of the core genome of the remaining 423 isolates. The average percentage of different core SNVs between genomes within each cgMLST cluster was then compared to the mean distance of identically sized random samples drawn from the total population. After 9999 iterations of Monte Carlo sampling, the top six clusters defined by cgMLST at *k*=5 grouped genomes together with a significantly higher similarity (average within-cluster SNV distance=0.002, 95 % CI: 0.001–0.003) compared to identically sized randomly drawn pools (Table 4). To visually examine the agreement between the structure of the population derived by both cgMLST and core SNV methods, we compared tree topologies by superimposing the cgMLST cluster assignments from the *k*=5 clustering threshold onto a minimum spanning tree generated from the core genome SNVs (Fig. 5). In general, strong agreement of the population structure was observed between both methods by comparing the position of genomes from each cgMLST cluster residing close to one another on the core genome SNV tree. A final cgMLST minimum spanning tree using this threshold was then used for visual comparison between the *S*. Heidelberg population structure and the distribution of sampling origin related to poultry species and poultry production stage (Fig. 6). The population of 430 *S*. Heidelberg isolates contained several clusters with apparent associations to broiler chicken, turkey, and mixed avian and human samples. Interestingly, Cluster 1 (indicated in dark orange in Fig. 5), a primarily turkey-associated cluster, contained no samples from human origin suggesting a lack of transmission of Cluster 1 isolates between turkeys and humans; otherwise, isolates from human sources were widely distributed throughout the minimum spanning trees (Fig. 6a, b). None of the six largest clusters identified in our analysis were restricted to any one poultry production stage (Fig. 6c), though Cluster 20 contained predominantly isolates from hatchery samples and a single isolate from human clinical origin.

### Associations between AMR and isolate ecology

#### Screening results

The following acquired resistance determinants and plasmid replicon types met our inclusion criteria of presence in 10–90 % of our sample for consideration as dependent variables for our multi-level models: the acquired resistance determinant bla_CMY-2_ with a total frequency of 36.3 % in our dataset (Table 2); and the plasmid replicon types ColRNAI and IncI1, with frequencies of 77.7 and 46.0% respectively (Table 3). According to the Phi correlation statistic, the IncI1 plasmid was significantly and strongly correlated with bla_CMY-2_ (Phi=77.8 %, *P* <0.05) and statistical results were the same for both determinants so only results for *bla*_CMY-2_ were presented. Other determinants identified using WGS were excluded from statistical models because they fell outside of the inclusion criteria.

#### Statistical model results

Univariable models were investigated for the outcomes of: (1) carriage of blaCMY-2 and (2) presence of ColRNAI plasmid type in accordance with our causal diagram in Fig. 2, and conceptual understanding of the intersection between stages of sampling (Fig. 1). Among poultry isolates, we observed a significant association between carriage of blaCMY-2 and Bird Species, with isolates from Chicken sources having increased odds for carriage of blaCMY-2 compared to those from Turkey (Table 5). No significant association between Production Stage and blaCMY-2 carriage was observed (Table 5). A significant association was observed for carriage of blaCMY-2 and cgMLST Subtype, with clusters 4, 18, 22, and 27 all having increased odds of carriage of blaCMY-2 compared to the referent (Table 5). The presence of ColRNAI among poultry isolates was significantly associated with Production Stage (Table 5), with isolates from Retail and Diagnostic samples having greater odds of ColRNAI present than the referent (Farm), respectively (Table 5). Bird Species was not found to be significantly associated with presence of ColRNAI (Table 5). The presence of ColRNAI was found to be significantly associated with cgMLST Subtype, with clusters 4 and 27 having increased odds for presence of ColRNAI compared to the referent (Cluster 1), respectively (Table 5).

**Table 5.** Odds ratio results for univariable multilevel models assessing the effects of poultry production stage, bird species and cgMLST subtype with the carriage of the *bla*_CMY-2_ acquired resistance gene and the ColRNAI plasmid replicon type for *Salmonella* Heidelberg isolates collected from poultry and human sources in Ontario, Canada, 2013

		*bla*_CMY-2_			ColRNAI		
		**OR**	(**95 % CI**)	**p**	**OR**	(**95 % CI**)	**p**
Production stage†*	(**Overall**)	–	–	**0.108**	–	–	**0.027**
	Farm	(Referent)	–	–	(Referent)	–	–
	Hatchery	0.17	(0.03–0.99)	0.049	0.43	(0.18–1.02)	0.056
	Abattoir	0.04	(0.00–3.41)	0.160	0.51	(0.09–2.79)	0.439
	Retail	0.61	(0.15–2.55)	0.502	1.58	(0.65–3.85)	0.309
	Diagnostic/Clinical	0.04	(0.00–0.73)	0.030	2.57	(0.53–12.49)	0.241
	*Variance (Establishment):*	*9.99*	*(4.8–20.9*)		*1.33*	*(0.4–4.1*)	
	*ICC (%):*	*75.2%*	*(59.3–86.4 %*)	*< 0.0001*	*28.8 %*	*(11.7–55.3 %*)	*0.001*
**Bird species†**	(**Overall**)	–	–	**0.001**	–	–	**0.408**
	Turkeys	(Referent)		–	(Referent)	–	–
	Chickens	10.4	(2.47–44.1)	0.001	1.35	(0.66–2.76)	0.408
	*Variance (Establishment):*	*7.90*	*(3.6–17.6*)		*1.34*	*(0.4–4.1*)	
	*ICC (%):*	*70.6 %*	*(51.9–84.2 %*)	*< 0.0001*	*28.9 %*	*(11.8–55.4 %*)	*0.001*
**cgMLST subtype**	(**Overall**)	–	–	**0.002**	–	–	**< 0.0001**
	Cluster 1	(Referent)	–	–	(Referent)	–	–
	Cluster 4	8.72	(2.23–34.12)	0.002	43.25	(10.42–179.6)	< 0.0001
	Cluster 18	5.57	(1.26–24.58)	0.023	1.02	(0.43–2.47)	0.958
	Cluster 20#	–	–	–	0.72	(0.16–3.15)	0.657
	Cluster 22	179.80	(9.11–3548.7)	0.001	4.33	(0.58–32.27)	0.153
	Cluster 27	22.44	(3.84–131.3)	0.001	54.52	(5.29–561.4)	0.001
	*Variance (Establishment):*	*4.22*	*(1.4–13.1*)		*1.11*	*(0.3–4.8*)	
	*ICC (%):*	*56.2 %*	*(29.2–79.9 %*)	*< 0.0001*	*27.5 %*	*(7.2 %–*65.0 %)	*0.07*

* Effect rendered non-significant with the addition of Bird Species in the model

**†** These models excluded isolates sampled from human origin

# Cluster 20 omitted due to complete presence of *bla*_CMY-2_

We attempted to construct multivariable models using the relationships described in our causal diagram (Fig. 2). For the blaCMY-2 outcome, cgMLST Subtype was no longer significant when included in a model with Bird Species (*P*=0.663), and was therefore excluded from the final model. A multivariable model including cgMLST Subtype and Production Stage was investigated for association with the presence of ColRNAI; in the multivariable model, the Production Stage variable was found to no longer be significant (*P*=0.741) and did not confound the relationship between cgMLST Subtype and the outcome, and was therefore dropped from the final model.

Model contrasts revealed that among poultry isolates, those sampled from retail and diagnostic sources were at significantly greater odds of carrying ColRNAI compared to isolates representing hatchery samples (Table 6). Additionally, isolates from Cluster 4 were at significantly greater odds of carrying ColRNAI compared to isolates from Clusters 1, 18, 20 and 22, however, no significant difference in this carriage was seen between Clusters 4 and 27 (Table 6). The intra-class correlation coefficient (ICC) in *bla*_CMY-2_ among isolates from the same establishment level exceeded 50 % in all models estimated for this outcome, while it approached nearly 30 % for models predicting the presence of ColRNAI (Table 5). For all final models, no outliers were identified, and the BLUPs met model assumptions of normality and homoskedasticity.

**Table 6.** Contrasts from multilevel univariable models for the association with ColRNAI in *Salmonella* Heidelberg isolates collected from poultry and human sources in Ontario, Canada, 2013

Model	Contrast	OR	(95 % CI)	*P*
**ColRNAI vs. production stage**	Retail vs Hatchery	3.67	(1.52–8.86)	0.004
	Clinical Diagnostic vs Hatchery	5.96	(1.23–28.84)	0.027
	Broiler Farm vs Hatchery	2.31	(0.98–5.48)	0.056
	Clinical Diagnostic vs Abattoir	5.03	(0.60–42.54)	0.138
	Retail vs Abattoir	3.10	(0.57–16.81)	0.190
	Clinical Diagnostic vs Broiler Farm	2.57	(0.53–12.49)	0.241
	Retail vs Broiler Farm	1.58	(0.65–3.85)	0.309
	Abattoir vs Broiler Farm	0.51	(0.09–2.79)	0.439
	Clinical Diagnostic vs Retail	1.62	(0.34–7.73)	0.542
	Abattoir vs Hatchery	1.18	(0.22–6.31)	0.844
**ColRNAI vs. cgMLST subtype cluster**	Cluster 4 vs Cluster 1	43.25	(10.42–179.56)	<0.0001
	Cluster 4 vs Cluster 18	42.25	(10.67–167.20)	<0.0001
	Cluster 4 vs Cluster 20	60.51	(9.65–379.48)	<0.0001
	Cluster 27 vs Cluster 1	54.52	(5.29–561.40)	0.001
	Cluster 27 vs Cluster 18	53.25	(5.37–528.48)	0.001
	Cluster 27 vs Cluster 20	76.27	(5.68–1025.14)	0.001
	Cluster 4 vs Cluster 22	9.99	(1.19–83.47)	0.034
	Cluster 27 vs Cluster 22	12.59	(0.76–208.50)	0.077
	Cluster 22 vs Cluster 20	6.06	(0.60–61.24)	0.127
	Cluster 22 vs Cluster 18	4.23	(0.59–30.28)	0.151
	Cluster 22 vs Cluster 1	4.33	(0.58–32.27)	0.153
	Cluster 20 vs Cluster 18	0.70	(0.17–2.90)	0.621
	Cluster 20 vs Cluster 1	0.71	(0.16–3.15)	0.657
	Cluster 27 vs Cluster 4	1.26	(0.13–12.45)	0.843
	Cluster 18 vs Cluster 1	1.02	(0.42–2.47)	0.958

## DISCUSSION

Whole-genome sequencing offers the highest level of discriminatory power for measuring relatedness between bacterial genomes, while also providing DNA-based inference on the presence of genetic factors related to antimicrobial resistance, lateral gene transfer, and the evolutionary history of a bacterial population. In the past decade, it has become increasingly common for studies to assert a ‘genomic epidemiology’ paradigm by superimposing geographic or source metadata onto descriptive results from WGS in the form of dendrograms or similar visualisations. Recently, studies have begun applying an analytical epidemiology framework to measure associations between the epidemiology of a pathogen and its genomic features [42, 43]. By including considerations for sampling and controlling for confounding at the analysis stage, conclusions generated from results from bioinformatic and genomic analyses can be more readily extrapolated to the level of the target population [16]. For the current report, we used this framework to (i) investigate the population structure of *S*. Heidelberg, and (ii) explore factors related to poultry production and population structure that may confer increased risk for carriage of elements related to the transmission of AMR in *S*. Heidelberg from poultry production in Ontario, Canada.

Several methodologies currently exist for estimating bacterial population structure from WGS data. Methods based on single-nucleotide variants (SNV) in the core genome of isolates under comparison offer the highest resolution and may become the gold-standard when attempting to discriminate samples that may be related to outbreaks, for example. For the analysis of data from longer-term surveillance initiatives, however, this increased resolution may hinder our ability to detect associations from the data. At the highest level of resolution, each individual isolate is treated as a unique observation rather than as a member of a group of highly related isolates representing a cluster. For this reason, we chose to employ cgMLST, a gene-by-gene approach to population genomics that has rapidly gained acceptance in the analysis of WGS datasets from surveillance on the strength of several features that provide increased tractability for large-scale, multi-year studies [44–46]. Unlike SNV-based methods, which require comparison to a closely related reference genome for variant identification, cgMLST indexes allelic variation in conserved genes that are defined during schema creation, and a database of allelic variantsis updated as new alleles become available. Meanwhile, the availability of pipelines such as *chewBBACA* [11] has made it possible to develop novel cgMLST schema that target the subspecies level and provide increased resolution over a species-level schema when working with highly clonal bacteria such as *S*. Heidelberg. For example, the schema created for this study expands on the discriminatory power that would be available through a general *S. enterica* cgMLST schema through the addition of *S*. Heidelberg-specific loci (*n*=371). Our decision to create a cgMLST scheme based on all available genomes of *S*. Heidelberg (*n*=4509) rather than just our study sample (*n*=430) was based on the desire to develop a robust method applicable to a larger sample of *S*. Heidelberg in future studies. While a cgMLST scheme based on our sample of 430 genomes alone would increase the discriminatory power of the method, using all available *S*. Heidelberg ensures that the same method can be applied to other samples in the future, and provide direct comparability to the results seen here.

Application of the novel *S*. Heidelberg-specific cgMLST schema to our dataset resulted in 334 clusters from a sample of 430 genomes when applying a distance threshold of 0 allelic differences for cluster definition. It was therefore necessary to identify distance thresholds that would generate clusters large enough to maintain statistical power in our analytical models, yet specific enough to only allow highly related observations to group together. We performed an analysis of cluster stability by recalculating the SID produced at each clustering threshold; three distinct peaks were observed where a single change to the clustering threshold resulted in a dramatic shift in SID and cluster membership. For our modelling analysis, we aimed to keep cluster sizes as large as possible while maintaining high discriminatory power and without prompting one of these major shifts in SID; we therefore chose the cluster designations defined at a threshold of five loci differences (i.e., *k*=5). In doing so, we maintained a similar population structure to the most discriminatory WGS-based thresholds (i.e., *k*=0–4), while ensuring that the cluster sizes generated by our cgMLST approach would be large enough to provide adequate power for statistical modelling. We then used the SNV profiles from each genome to assess the degree of genomic similarity within and between each of the six largest cgMLST clusters beyond that of chance using a Monte Carlo hypothesis testing approach. Combined with our assessment of population cluster stability, we believe these approaches may provide a systematic framework for future efforts in establishing optimal clustering thresholds for the analysis of WGS data from infectious disease surveillance.

Results from our analysis of the population structure of *S*. Heidelberg implicate several strains in circulation in both poultry and human sources. Of the six clusters analysed for this study, only Cluster 1 appeared to be restricted uniquely to poultry sources; the remaining five clusters contained isolates from both poultry (predominantly chicken) and human isolates, demonstrating the potential for transmission between these sources (Fig. 6a–c). The *bla*_CMY-2_ beta-lactamase gene was observed in isolates from each of these clusters – suggesting that poultry contributes to the human exposure with this clinically relevant AMR determinant, with significantly higher odds of carriage in chicken isolates compared to turkey (Table 5). Interestingly, however, only isolates from Cluster 20 were consistently positive for both IncI1 and *bla*_CMY-2_; isolates positive for conjugative IncI1 from the remaining five clusters were both positive and negative for the *bla*_CMY-2_ gene. Taken together, the distribution of isolates both positive and negative for the *bla*_CMY-2_ allele and the IncI1 plasmid throughout several highly clonal clusters of *S*. Heidelberg associated with both poultry and human sources is further evidence of a highly promiscuous plasmid group circulating throughout several distinct strains of *S*. Heidelberg, and not the result of clonal expansion by a single resistant bacterial subpopulation; this observation is similar to what has been previously reported for Canadian *bla*_CMY-2_ *S*. Heidelberg from Quebec, Canada [21].

We observed the *fosA7* gene in almost all isolates included in this study (*n*=423/430). This gene was previously reported in all *S*. Heidelberg sampled from broiler chickens in British Columbia, Canada and was shown to provide complete resistance to fosfomycin when located either on the chromosome or in a plasmid vector [47]. Fosfomycin was recently approved for use in human clinical practice in Canada to treat bladder complications such as lower urinary tract infection [48] and has been reported as an effective agent for treating infectious diseases in broilers and swine in Central and South America [49]. The detection of *fosA7* in such high frequency among *S*. Heidelberg genomes from Ontario in our study suggests the potential for widespread dissemination of fosfomycin resistance among *S*. Heidelberg in Canada, although its use in veterinary medicine in Canada appears limited to domestic animals [49, 50]. Further surveillance and study of this gene is necessary to determine its origin and persistence in *S*. Heidelberg from poultry production in Canada.

The AmpC beta-lactamase gene *bla*_CMY-2_ has previously been identified as a major determinant for third-generation cephalosporin resistance in *Salmonella enterica* subsp. *enterica* and is thought to have spread throughout *Salmonella* and *E. coli* populations in North America by its location on highly mobile, conjugative plasmids [51]. Our results support this hypothesis, with a significant positive correlation between the presence of a conjugative IncI1 plasmid and *bla*_CMY-2_. Of note, however, are several isolates (*n*=46/430) that when queried using *in silico* methods, possessed the IncI1 plasmid but not *bla*_CMY-2_. While this discrepancy may be due in part to error inherent in the sequencing, assembly, and AMR prediction stages of the bioinformatic analysis, it seems more likely that these isolates in fact harbour IncI1 plasmids that differ significantly in their genomic content. These results, along with those seen previously [21] suggest that plasmids possessing the IncI1 replicon type may represent a diverse population in Canadian *S*. Heidelberg, and that a separate analysis of the distribution of variants of this important plasmid is warranted to further characterize the transmission dynamics of AMR related to this plasmid throughout *Salmonella* in Canadian poultry production.

Results from our modelling analysis on *S*. Heidelberg from Ontario poultry samples indicate a significant association between bird species and the carriage of *bla*_CMY-2_, with odds of *bla*_CMY-2_ carriage in isolates sampled from chicken sources significantly greater than those from turkey sources. Interestingly, the stage of poultry production from which isolates were sampled had no such association with the presence of *bla*_CMY-2_, suggesting that by the time our samples were collected (2013), *bla*_CMY-2_ had already experienced widespread dissemination throughout *S*. Heidelberg in Ontario broiler production, perhaps due to an earlier introduction in chicken than in turkey farming environments, or differences in selective pressures (e.g., on-farm use of antibiotics) [19]. A contrasting relationship was observed for the presence of the ColRNAI plasmid replicon type; bird species had no significant association with ColRNAI, however, later stages in production showed higher odds of ColRNAI presence compared to earlier stages (Table 6; e.g., Retail vs. Hatchery and Clinical Diagnostic vs. Hatchery). The higher odds of ColRNAI presence at later stages of production may be the result of this plasmid conferring a selective advantage to *S*. Heidelberg strains via increased fitness and survival capabilities along the poultry production continuum (e.g., increased mobility, adherence, oxidative stress and/or temperature tolerance, resistance to disinfection). Previous work has demonstrated the presence of Col-type plasmids conferring a selective fitness advantage to *S*. Heidelberg in poultry litter [52]. While it is beyond the scope of the current analysis, further work should investigate whether genetic factors may be present in this plasmid family that allow for increased survival along the poultry production continuum.

Human isolates in this study were collected independently as part of routine provincial notifiable disease surveillance. As these submissions may be the result of non-poultry exposures, we excluded them from models assessing the association with poultry production stage and bird species as these data were collected specifically through active and passive surveillance of poultry production. We did, however, include human isolates in our models related to cgMLST clusters as the assignment of isolates to these groupings should, in our opinion, be independent of sample collection, or at least indicate genetic clusters associated with human cases. Unconditional univariable models for both *bla*_CMY-2_ and ColRNAI indicated a significant association with the major cgMLST subtypes identified in our analysis of *S*. Heidelberg population structure (Table 5). For example, Clusters 4, 18, 22, and 27 were all significantly associated with higher carriage of the bla_CMY-2_ gene (Table 5), and Clusters 4 and 27 were at significantly higher odds of carriage for the ColRNAI plasmid (Table 6), suggesting that application of genome-based subtyping approaches may be useful to identify sub-lineages within the highly clonal *S*. Heidelberg population that pose a higher risk to human health through the carriage of genes and plasmids related to antimicrobial resistance. These modelling results should be interpreted with caution, however, due to the wide confidence intervals present in many of the odds-ratios for cgMLST clusters, which are likely due to the difference in the size of membership between each of the clusters (Table 4). While the fit of these models was assessed based on assumptions of normality and homogeneity of variance of the BLUPs, a larger sample including more isolates per cgMLST cluster would likely result in narrower confidence intervals for the estimated odds ratios. The general observation, however, that some cgMLST clusters are consistently more likely to carry genetic elements responsible for resistance to antimicrobials important to human medicine still holds. Identifying these subpopulations is important for surveillance of AMR in the farm-to-fork continuum as they may be more likely to contribute to horizontal gene transmission events and the overall spread of AMR through food systems.

Another potential limitation of this study is that the selection of isolates for WGS comprised a subset from a retrospective collection of *S*. Heidelberg isolates sampled via national surveillance and baseline programs. While the original sampling plans for the surveillance initiatives were designed to enable comparisons between the different nodes of poultry production (e.g., on-farm, abattoir, and retail components), the selection of isolates for treatment by WGS was done using a convenient selection process to ensure adequate power for comparisons in other analyses (e.g., future work describing the temporal and geographical trends of AMR in *Salmonella* from poultry production at the national level). As is the case in any study employing convenience-based sampling, this may limit the external validity of our results; multi-stage random sampling from the isolate population would have been the preferred approach. Thus, although the original WGS dataset comprised over 2400 *S*. Heidelberg genomes from across Canada, we decided to focus our analysis on isolates exclusively from poultry production and human sources from Ontario in 2013 in order to minimize the confounding effects of inconsistent sampling across province and time. Although this led to a significant reduction in the number of available genomes suitable for inclusion into the study, it allowed us to avoid confounding effects of inconsistent sampling across province and year and strengthened our ability to extrapolate results to the provincial level. We controlled for autocorrelation among isolates collected at the same sampling event by the inclusion of a random intercept for ‘establishment’ in our models. The variance estimate listed in Table 5 (e.g., ‘Variance [Establishment]’) refers to the variance estimated at the establishment level since this multilevel model partitions the variance in the outcome at the individual isolate-level and establishment-level; in the case of multilevel logistic regression models, the variance at the individual level can be approximated using the latent variable technique [16, 41]. This is closely tied to the intra-class correlation coefficient (ICC) (Table 5), which uses the variance component at the establishment level divided by the total variance in a two-level model to estimate the correlation between observations within the same establishment after accounting for the fixed effects in the model. The high values for variance and ICC (Table 5) in five out of six of our final multilevel logistic regression models suggest that isolates sampled from within the same establishment cluster were strongly correlated and should not be treated as independent units in statistical analyses. Rather, the application of multilevel models including random effects, as applied in the current study, should be used to control for this autocorrelation. The increasing adoption of routine whole-genome sequencing for isolates from surveillance and baseline programs is resulting in a critical mass of genomic data that should permit the application of analytical epidemiology approaches that have been demonstrated here to complement existing tools and approaches in genomic epidemiology. In turn, this will facilitate the transition from a paradigm in which genomic epidemiology is primarily used as a tool to investigate specific public health events towards the translation of genomic surveillance data into public health policy.

## Supplementary Data

Supplementary material 1
